# The impact of the COVID-19 pandemic on patterns of attendance at emergency departments in two large London hospitals: an observational study

**DOI:** 10.1186/s12913-021-07008-9

**Published:** 2021-09-23

**Authors:** Michaela A. C. Vollmer, Sreejith Radhakrishnan, Mara D. Kont, Seth Flaxman, Samir Bhatt, Ceire Costelloe, Kate Honeyford, Paul Aylin, Graham Cooke, Julian Redhead, Alison Sanders, Helen Mangan, Peter J. White, Neil Ferguson, Katharina Hauck, Shevanthi Nayagam, Pablo N. Perez-Guzman

**Affiliations:** 1 MRC Centre for Global Infectious Disease Analysis, Department of Infectious Disease Epidemiology, Imperial College London, Norfolk Place, London, W2 1PG UK; 2 Modelling and Economics Unit, National Infection Service, Public Health England, 61 Colindale Avenue, London, NW9 5EQ UK; 3 NIHR Health Protection Research Unit in Modelling and Health Economics, Imperial College London, Norfolk Place, London, W2 1PG UK; 4 Department of Mathematics, Imperial College London, South Kensington Campus, London, SW7 2AZ UK; 5 Section of Epidemiology, Department of Public Health, University of Copenhagen, Copenhagen, Denmark; 6 Imperial College London Department of Primary Care and Public Health, Global Digital Health Unit, St Dunstan’s Rd, Hammersmith, London, W6 8RP UK; 7 Imperial College Healthcare NHS Trust, The Bays, South Wharf Road, London, W2 1NY UK; 8 West London Mental Health NHS Trust, 1 Armstrong Way, Southall, London, UB2 4SD UK

**Keywords:** COVID-19, Emergency department, Accident, And emergency

## Abstract

**Background:**

Hospitals in England have undergone considerable change to address the surge in demand imposed by the COVID-19 pandemic. The impact of this on emergency department (ED) attendances is unknown, especially for non-COVID-19 related emergencies.

**Methods:**

This analysis is an observational study of ED attendances at the Imperial College Healthcare NHS Trust (ICHNT). We calibrated auto-regressive integrated moving average time-series models of ED attendances using historic (2015–2019) data. Forecasted trends were compared to present year ICHNT data for the period between March 12, 2020 (when England implemented the first COVID-19 public health measure) and May 31, 2020. We compared ICHTN trends with publicly available regional and national data. Lastly, we compared hospital admissions made via the ED and in-hospital mortality at ICHNT during the present year to the historic 5-year average.

**Results:**

ED attendances at ICHNT decreased by 35% during the period after the first lockdown was imposed on March 12, 2020 and before May 31, 2020, reflecting broader trends seen for ED attendances across all England regions, which fell by approximately 50% for the same time frame. For ICHNT, the decrease in attendances was mainly amongst those aged < 65 years and those arriving by their own means (e.g. personal or public transport) and not correlated with any of the spatial dependencies analysed such as increasing distance from postcode of residence to the hospital. Emergency admissions of patients without COVID-19 after March 12, 2020 fell by 48%; we did not observe a significant change to the crude mortality risk in patients without COVID-19 (RR 1.13, 95%CI 0.94–1.37, *p* = 0.19).

**Conclusions:**

Our study findings reflect broader trends seen across England and give an indication how emergency healthcare seeking has drastically changed. At ICHNT, we find that a larger proportion arrived by ambulance and that hospitalisation outcomes of patients without COVID-19 did not differ from previous years. The extent to which these findings relate to ED avoidance behaviours compared to having sought alternative emergency health services outside of hospital remains unknown. National analyses and strategies to streamline emergency services in England going forward are urgently needed.

**Supplementary Information:**

The online version contains supplementary material available at 10.1186/s12913-021-07008-9.

## Background

In March 2020, the World Health Organization declared the outbreak of COVID-19 as a pandemic [1]. To tackle it, the United Kingdom (UK) Government instituted fundamental changes to the provision of health and social services [2, 3]. As a result, the National Health Service (NHS) undertook an unprecedented re-arrangement of resources, with specific measures including the postponing of non-urgent elective procedures, and provision of video consultations of patients in the community for referral to hospital services [2]. Moreover, on March 12, 2020 the UK Government implemented the first of a series of non-pharmaceutical interventions, including advice for the public to self-isolate if experiencing COVID-19 symptoms, advice for social distancing, the closure of schools and universities and the ban of public events [2]. These measures were rapidly followed by a national lockdown on March 24 and legislation indicating people to stay at home and avoid social interaction with others outside their households, unless an emergency arises [2, 4].

Largely as a result of the widespread implementation of such non-pharmaceutical interventions in the UK, a steady reduction in the daily number of COVID-19 cases and deaths was observed between April and August, 2020 [4–7]. Despite this, the number of attendances to emergency departments (ED) (i.e., consultant-led, 24-h services including resuscitation units) decreased by approximately 50% across England and remained low during the same period of time [8, 9]. Whilst it has been reported that the reduction in ED attendances was mainly seen for low acuity cases [9], attendances also reduced significantly in key disease areas such as life-threatening surgical emergencies and acute coronary syndromes which may have led to an increase in out-of-hospital deaths [10–12].

Outside the UK, evidence from Italy and France indicates that the number of out-of-hospital cardiac arrests increased alongside a decrease in ED attendances during the first wave of the COVID-19 pandemic [13, 14]. These data also suggest that the number of attendances without COVID-19 attendances to emergency services did not increase as expected as COVID-19 cases and deaths decrease [13].

Beyond high-level national trends, analyses of sociodemographic factors leading to reductions in attendances without COVID-19 ED attendances are crucial to understand the intended and unintended implications of reconfiguring emergency care resources. Such analyses can help better inform a public health response to revert these trends and ensure continued high-quality standards of care for patients without COVID-19, whilst ensuring ED services do not revert to the overcrowding seen prior to the pandemic [15, 16].

In this study, we use pseudonymised administrative patient-level records from Imperial College Healthcare NHS Trust (ICHNT) to: a) analyse local trends and factors associated with ED attendances and emergency admissions pre- and post-implementation of lockdown policies on March 12, 2020 in England; and b) analyse regional (all London ED services) and national situation reports to understand the magnitude and directionality of how our local trends compare against these.

## Methods

We conducted an observational study of ED attendances to two London hospitals. We accessed to historical (2015 to 2019) and present year (January 1 to May 31, 2020) pseudonymised data on: a) ED attendances to St Mary’s and Charing Cross Hospitals compromising all ED attendances to ICHNT and b) hospital admissions to these two and other hospitals of ICHNT (Queen Charlotte’s and Chelsea Hospital, Hammersmith Hospital and Western Eye Hospital), one of the largest NHS Trusts in England and one serving a population with a higher than national proportion of ethnic minorities of all ages [17, 18]. St Mary’s Hospital (SMH) is the major trauma centre for North West London with a major trauma centre. Charing Cross Hospital (CXH) is a major acute hospital including a hyper acute stroke unit. Before the start of the pandemic, these EDs were seeing an average of 208 [min 99, max 289] and 106 [min 60, max 177] patients of all ages, respectively, per day [19].

We defined two periods of interest: the period from January 1 till March 11, 2020 and March 12 till May 31, 2020. This was based on the data when the first public health measure (case-based isolation) leading to lockdown was imposed in England on March 12, 2020 [2]. We accessed historic data of daily ED attendances starting on April 1, 2019 and used it to calibrate a time series forecast model to predict the expected number of ED attendances as a counterfactual for the time period from March 12 to May 31, 2020. The forecasted trend was compared against observed ED attendances in that same period. Forecasts time series were produced using Auto Regressive Integrated Moving Average (ARIMA) models. These simple stochastic time series models capture temporal structures within historic time series datasets and can thus be used to forecast future values [20]. The ARIMA model consists of three parts including an autoregressive component (AR), an integrated part (I) and a moving average (MA) component. The autoregressive part indicates that the variable of interest, in our case the number of daily attendances, is regressed on its own previous past values of demand. The integrated component refers to several differencing steps making the time series stationary while the moving average component indicates that the regression error is a linear combination of past error terms. More details of the ARIMA model’s construction can be found in the Supplement. The validity of these models in predicting demand in ED attendances has been previously validated by authors in our group [19].

To build a regional and national scenario against which to compare the overall trends of ED attendances and emergency admissions to ICHNT, we accessed publicly available monthly NHS England situation reports [8]. Data between June 2015 and December 2019 were used to parameterise ARIMA models by region (London, Midlands, North and South) and nationally and forecast expected ED attendances and emergency admissions from January to May 2020, which we compared to data from situation reports for the same period.

For the case of hospital admissions to ICHNT, we further analysed data on admissions that were from amongst ED attendances between January 1 and May 31. Data from this period in historic records from the past five years, 2015–2019, was used to average the number of emergency admissions and compare against observed data for the same time period in 2020.

Our primary outcome of interest was the percent change in observed vs expected daily ED attendances and emergency admissions to ICHNT post-March 12, 2020. We used a general linear regression model to assess the effect of selected variables on the number of ED attendances. These included the distance from the postcode area of residence to the hospitals, the population-weighted index of multiple deprivation (IMD) quintile and the mean number of historic attendances. The IMD and the population (for weighting) data was obtained from publicly available resources from the Office for National Statistics and the Ministry of Housing, Communities and Local Government at the lower layer super output areas [21, 22], which we aggregated to outer postcodes. To ensure anonymity, only the patients’ postcode area (i.e., first two to four alphanumeric characters) was used and then aggregated into five mutually exclusive zones, based on the distance of the centroid of the postcode area to the hospital of attendance:

• Zone A, less than 1000 m.

• Zone B, between 1001 m and 5000 m.

• Zone C, between 5001 m and 7500 m.

• Zone D, between 7501 m and 10,000 m.

• Outer zone, greater than 10,000 m.

Additional outcomes of interest were the change in:

• Time series of ED attendances by age, sex, mode of arrival (e.g., ambulance, own transport or other) and zone of residence (see Figs. S3, Fig. S4 and Table S4c in the Supplement).

• Emergency admissions by disease categories, as per ICD-10 codes (see Table S6 in the Supplement).

• Overall and disease area-specific mortality risk ratio amongst emergency admissions.

(see Table S6 in the Supplement).

All statistical and geo-spatial analyses were performed in R 3.6.3, with the latter using freely available polygon files [23].

### COVID-19 diagnosis criteria

As previously published from our group and following institutional policies at the time [24], all patients presenting with respiratory symptoms at ICHNT were tested for a SARS-CoV-2 PCR. As per institutional policies at the time, an ICD-10 diagnosis of COVID-19 was recorded for discharged or deceased patients either: 1) on the date of collection for a sample positive for SARS-CoV-2 PCR (even retrospectively); or 2) when a clinical suspicion of COVID-19, as documented by the treating medical team (in medical notes, discharge documents or death certificate) was present despite a negative PCR.

### Study approval and role of the funding sources

To ensure compliance with General Data Protection Regulations, data was extracted from pseudonymised datasets into aggregate reports for the outcomes of interest. Data processing was authorised by both the ICHNT and School of Public Health research committees and jointly granted by the Trust’s Data Protection Office, Caldicott Guardian, Medical Director and the College’s Big Data and Analytical Unit, under Article 6 (1)(e) / 9 (2)(i) of the General Data Processing Regulations (processing under public authority for purposes in the area of public health). Funders had no role in the study design, data collection, analysis, interpretation, or reporting.

## Results

### Overall observed vs forecasted ED attendances

**Fig. 1:**
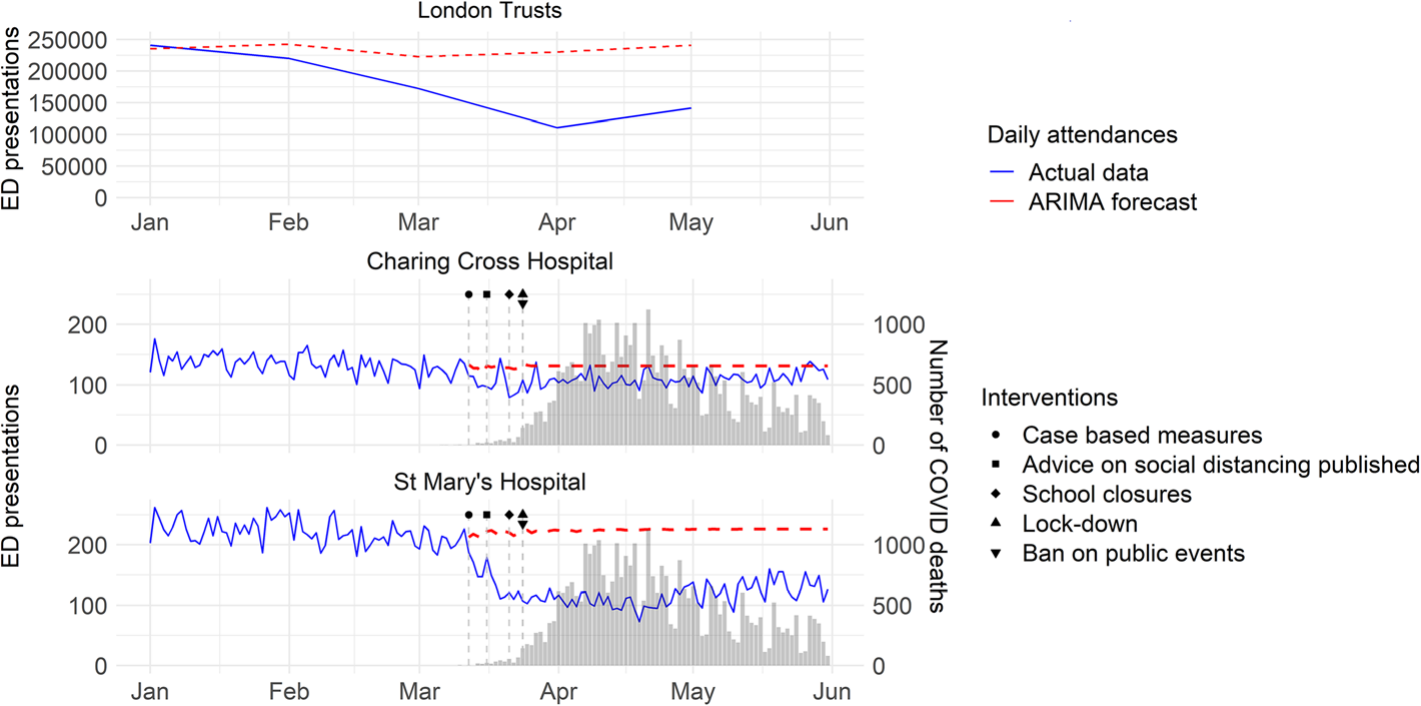
Time series of attendances to ED services at ICHNT (Charing Cross Hospital and St Mary’s Hospital) in relation to the regional (all London EDs) decline in attendances. Data for COVID-19 deaths in background bar chart as collated from daily Public Health England reports into publicly available repository, available at: https://raw.githubusercontent.com/tomwhite/covid-19-uk-data/master/data/covid-19-totals-england.csv

Between January 1 and March 11, 2020 there were 25,203 total attendances to ED services at ICHNT, which fell within the forecasted number of attendances (26,396, 95%CI 8571 to 44,221). After March 12, however, we observed a significant decline in the number of attendances, amounting to 18,569 as of May 31,2020. This represented a 35% decline against our forecast (28,774, 95%CI 26,625 to 30,923) (Fig. 1).

The overall decline in ED attendances to ICHNT was largely in keeping with the national trend which dropped by 49.3% in April 2020 compared to the mean prediction resulting from an ARIMA forecast during the start of the current COVID-19 pandemic response (or 46.3 to 51.9% when compared to the predicted 95%CI) - see also Fig. 1 and Supplementary Fig. S2. However, for ICHNT the observed trend was mainly driven by a reduction in attendances to SMH, which dropped by 46% compared to the mean prediction (or 42 to 50% when compared to the predicted 95%CI) compared to only 17% decrease with respect to the mean prediction (or 11 to 22% when compared to the predicted 95%CI) for CXH (Fig. 1).

### Disaggregated trends in ED attendances to ICHNT

From the start of the year 2020 to March 11, the number of daily ED attendances by age to ICHNT comprised mainly of people aged 22 to 64 years, followed by those older than 65 years and paediatric attendances, in line with historic trends. Between March 12 and May 31, 2020, we observed a much larger decline in attendances amongst younger age groups, compared to those over 65 years, particularly for the case of SMH (Supplementary Fig. S3a and Table S2).

Throughout the period of January 1 to May 31, 2020, the predominant mode of arrival to ICHNT for ED attendances was by patients’ own transport, followed by road ambulance services. However, after March 12 there was a significant drop in the former, which was superseded by a proportional increase in ambulance arrivals (Supplementary Fig. S3c and Table S4). Importantly, these changes were driven by attendances to SMH alone, as the proportional distribution of attendances at CXH by arrival mode did not vary significantly before and after March 12, 2020.

**Fig. 2:**
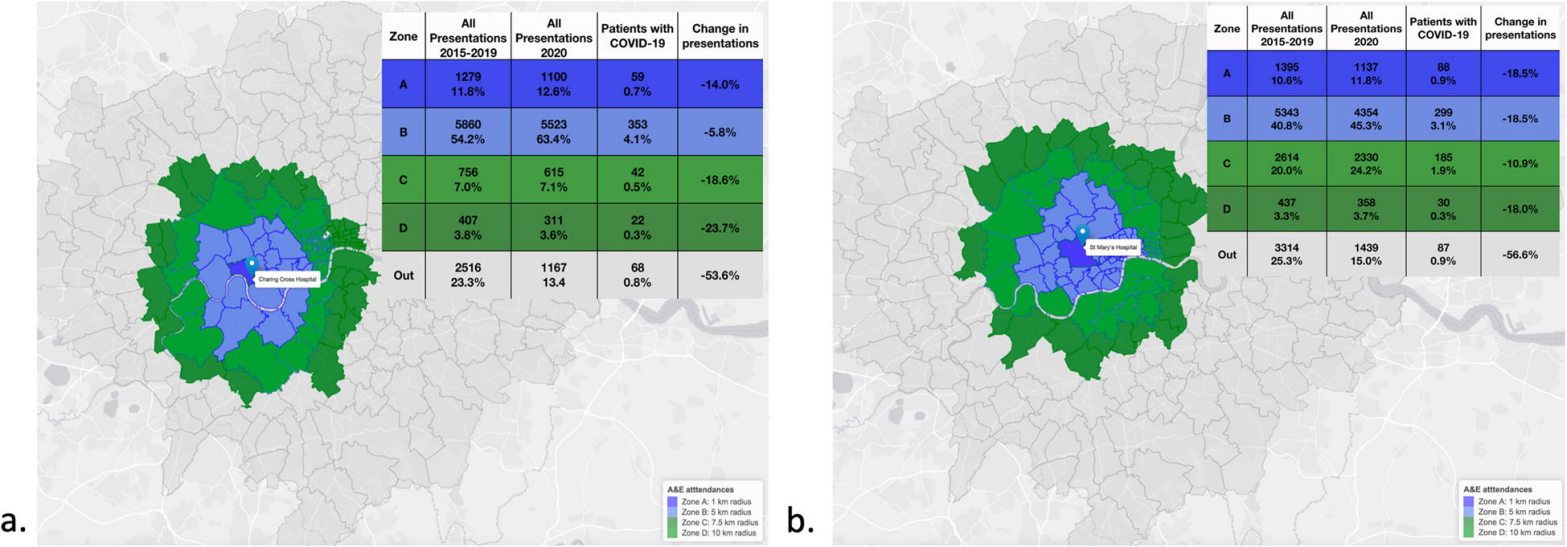
Attendances by geographic area of patient residence to (**a**) Charing Cross and (**b**) St Mary’s hospitals between March 12 and May 31. The maps show A (< 1000 m), B (1001 m–5000 m), C (5001 m–7500 m), D (7501 m-10,000 m), and Outer (> 10,000 m) zones defined as the distance from the centroid of the postcode to hospital. Percentages in the “All presentations” columns refer to total presentations in the respective column. Percentages in the “Patients with COVID-19” columns refer to the proportion of all attendances that became COVID-19 hospitalisations. Percentages in the “Change in presentations” columns refer to the difference in all ED attendances in 2020 compared to historic (2015–2019) data. This figure has been created using R version 4.0.3

Additionally, we observed significant differences in the number of attendances to each hospital by zone of patients’ postcode of residence (as defined in methods) (Fig. 2). The percent distribution of attendances by zone of residence did not differ significantly when comparing the pre- and post-March 12, 2020 periods (*p* = 0.99) and neither did the distribution of attendances by increasing distance between patients’ postcode of residence to the hospital. It was also not explained by population-weighted IMD quintile (Supplementary Table S5).

### Emergency admissions and outcomes by disease area

We recorded a total of 16,837 emergency admissions to ICHNT between January 1 and May 31, 2020 and compared these to the average number of admissions for the same period of time over the previous five calendar years. The largest drop in admissions was seen for the period between March 12, 2020 and May 31, 2020 at 39% (6545), compared to 14% (10,292) in the period between January 1, 2020 and March 11, 2020.

**Fig. 3:**
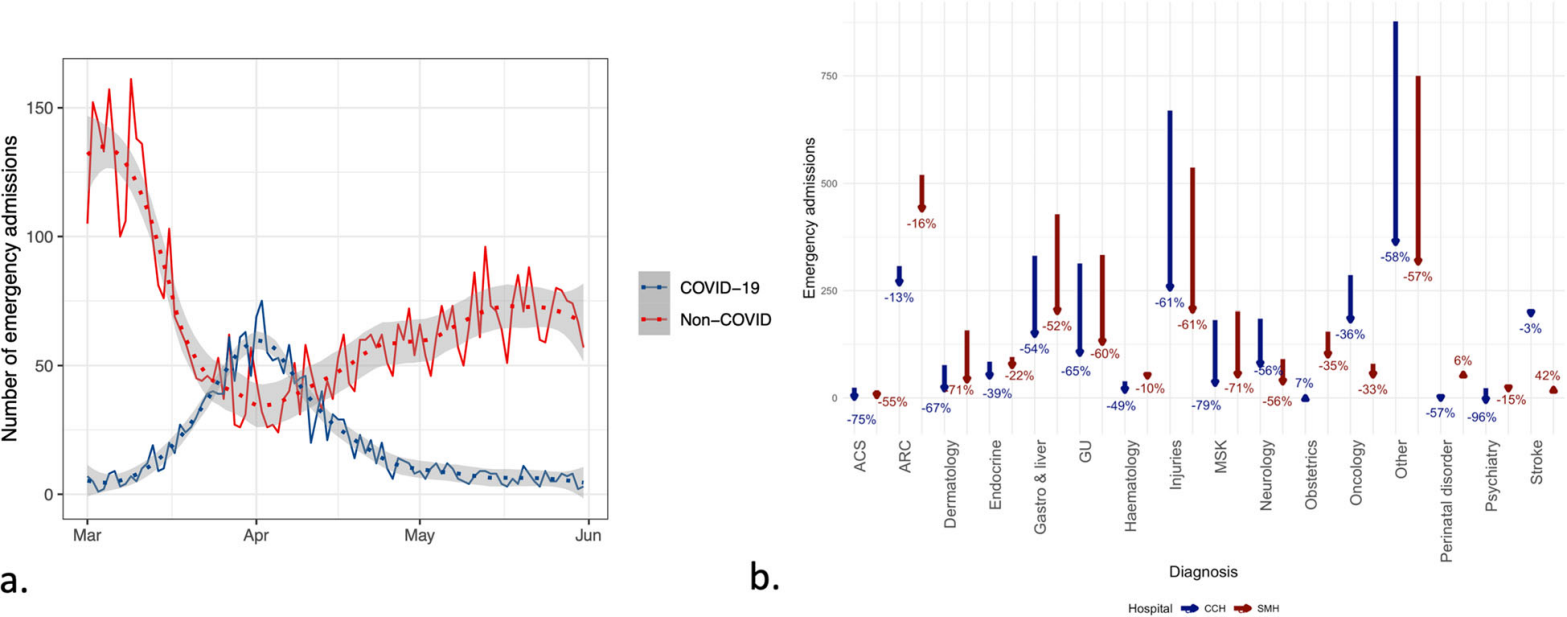
Daily emergency admissions at ICHNT of (**a**) non-COVID vs COVID-19 patients and (**b**) change by disease area for the period of March 12 to May 31, 2020 compared to historic (2015–2019) average. In (**b**), the absolute changes for Charing Cross (blue) and St Mary’s (red) hospitals are shown by arrows, with labels showing percentage changes. The historic data refers to the average of emergency admissions between the period from March 12 and May 31 for each year. (ACS – Acute Coronary Syndrome, ARC – Acute Respiratory Conditions, GU – Genito-Urinary conditions, MSK – musculoskeletal)

Out of all emergency admissions, COVID-19 was either the cause or a co-factor (i.e. infection documented either at admission or during hospitalisation, respectively) for admission in 1408 (8%) patients between January 1 and May 31, 2020. All but three of these COVID-19 admissions occurred after March 12, 2020 (21% of admissions after this date were related to COVID-19) (Fig. 3a). Therefore, when excluding admissions related to COVID-19 after March 12, 2020 we saw that the actual reduction in emergency admissions without COVID-19 was 48% (5171) compared to the average over the same period in the past five years (10,772).

During the time period of March 12 and May 31, 2020, most emergency admissions without COVID-19 were for acute respiratory conditions (*n* = 802, 12%), including pneumonia, asthma and chronic obstructive pulmonary disease exacerbations, amongst others; injuries (*n* = 540, 8%); gastrointestinal and liver disorders (*n* = 372, 6%); and genitourinary disorders (*n* = 315, 5%).

In accordance to the overall trend, for most disease areas and even for critical ones we saw a decrease in admissions (Fig. 3b). For example, acute coronary syndromes and stroke admissions decreased by 60 and 26%, respectively. Obstetric and perinatal emergency admissions also declined by 52 and 24%, respectively. Lastly, in key disease areas for which ICHNT is a referral centre, such as cancer-related emergency admissions (i.e. excluding programmed interventions and/or procedures, like chemo- and radiotherapy) and those due to injuries also dropped by 47 and 64%, respectively.

Whilst the crude in-hospital mortality for emergency admissions for the period between March 12 and May 31 increased from 1% historically (2015–2019) to 8% in 2020 (incidence risk ratio [IRR] 2.84, 95%CI 2.48–3.26, *p* < 0.001), this was driven by deaths relating to COVID-19 (Supplementary Table S6). When excluding these deaths from analyses, we saw the crude mortality risk for emergency admissions without COVID-19 was not significantly different from historic trends (IRR 1.13, 95%CI 0.94–1.37, *p* = 0.19). In fact, for most specific disease areas, we saw an overall reduction in mortality risk (Supplementary Table S6), including acute respiratory conditions, acute coronary syndromes, oncological emergencies and injuries.

## Discussion

The current COVID-19 pandemic has created unprecedented challenges for emergency health services in England. Our study is the first to analyse detailed trends of ED attendances to one of the largest NHS trusts in England. We use ICHNT as a case study to compare it to regional and national trends. We find that overall ED attendances decreased by 35% at ICHNT, which is comparatively lower than the trend for all London ED services and the whole of England (42 and 37%, respectively). Changes in transportation use and hospital avoidance behaviours are a potential key driver of these differences. When analysing disaggregated trends for ICHNT, we found evidence of additional factors associated with decreased ED attendances, such as age and acuity of presentation patterns. Our analyses have important and interrelated public health implications at a national level.

Firstly, we identified significant variation in the decrease of ED attendances between the trust, regional and national level. On the one hand, SMH is located in the central London borough of Westminster (next to a major railway station), which historically receives the largest inflow of daily commuters in the country [25], and CXH is located in a residential area of the borough of Hammersmith and Fulham. Attendances to the former dropped by 46%, compared to 17% to CXH. During the first wave of the pandemic, public transportation by railway and underground tube plummeted from 96% of expected travellers on March 12, 2020 to 4% on April 10, 2020 [26]. On the other hand, at a regional level, the decrease in ED attendances was more comparable, dropping to 50% for London and the South of England, 48% in the North and 52% in the Midlands by April 2020 (Supplementary Fig. S2). We did not find the distance from residence to hospital to be a predictor of reduced ED attendances at either ICHNT hospital. Overall mobility reduced during lockdown in England [27], with people thus avoiding travelling if possible and those experiencing an emergency may have avoided seeking care in hospital ED services altogether, a hypothesis that is supported by emerging data from population behavioural data during the COVID-19 pandemic [28].

Secondly, and added to the above, we saw that ED attendance patterns of patients aged > 65 years were not really affected. Younger age groups correspond to the main proportion of daily commuters [25]. This sector of the population is also more frequently suffering from injuries and trauma-related emergencies, which we found to have decreased by 64% despite SMH being a key trauma care pathway. Moreover, the structure of the two EDs at ICHNT is so that paediatric (< 18 years) emergencies are mostly managed at SMH, with virtually none seen at CXH. National public health advice during COVID-19 has greatly reduced mobility across the country to a level comparable to the one reached during weekends pre-March 2020 [19, 27]. On the other hand, those aged over 65 represent the age group most affected by COVID-19. At ICHNT, they have accounted for 56% of all COVID-19 admissions and 78% of deaths [18]. We found that, during late March, 2020, and early April, 2020, COVID-19 admissions in fact superseded all-cause emergency admissions without COVID-19 at our hospitals.

Thirdly, we saw a proportional increase in ED attendances arriving by ambulance services. While this could partially be due to less commuting as mentioned before, it could also be a marker for illness severity. Public concerns have been voiced during lockdown alerting that people experiencing an emergency may have delayed or avoided attending an ED. [29] Moreover, conclusive evidence has recently indicated that deaths of patients without COVID-19 in the community increased during lockdown, particularly amongst the elderly [10, 11, 30]. National level trends in England show some evidence that particular age and acuity of presentation groups of patients had different patterns of ED seeking behaviours, with paediatric attendances not delayed compared to pre-COVID-19 [30], and elderly or more sever groups having a lower reduction in number of attendances [9]. Yet the extent to which delayed and/or avoided ED attendance amongst older age groups is correlated to the observed increase in deaths of patients without COVID-19 in the community is still unknown and warrants urgent investigation.

Lastly, after removing COVID-19 admissions and deaths, we saw the crude mortality risk in patients without COVID-19 was not higher compared to historic trends (RR 1.13, 95%CI 0.94–1.37, *p* = 0.19). In parallel to the restructuring of emergency hospital services, ambulatory healthcare was also substantially changed to tackle increased demands during lockdown. This included implementing extended-hours practices, virtual general practice consultations, additional pathways for key disease areas, such as mental health, and expansion of telephone assessment services [31]. Such measures had an impact on the case mix of emergency admissions at ICHNT and may have also helped reduce pressure on ED services (i.e. by dealing with emergencies amenable to be managed in the community). Whether these or other factors meant mortality amongst emergency admissions at ICHNT was kept at its historical low levels or there was indeed an increase in mortality, which did not reach statistical significance due to sample power, is unknown. Nevertheless, the absolute positive effect of the above measures to provide out-of-hospital emergency care in England during lockdown warrants further investigation. Valuable lessons can be learnt, so that a sustainable streamlining of urgent and emergency care can be achieved going forward. Especially since rapidly scaling up capacity during the COVID-19 crisis has put already under-resourced areas of care in additional economic constraints [32], so their capability to respond to even greater demands going forward could be compromised.

Our study has limitations that must be acknowledged. Firstly, whilst we analysed publicly available monthly (aggregated) situation reports from all ED NHS Trusts in England, we only disaggregated these trends for the case of ICHNT in London. Whilst this is one of the largest NHS Trusts in England and a key referral pathway for major trauma, cancer care and respiratory and cardiovascular emergencies, further analysis and comparisons with other acute trusts are needed. Secondly, a change in administrative coding systems between our historic and present year datasets (i.e., between Secondary Uses Services [SUS] and Systematised Nomenclature of Medicine Clinical Terms [SNOMED] systems, respectively) limited our ability to compare the change in patients’ diagnoses on presentation to ED. We thus relied on aggregating hospital admissions by disease area, based on comparable ICD-10 codes between the two periods. By and large, the group of patients admitted to hospital from the ED represents those who are the sickest and thus warranted in-hospital stay and management.

## Conclusions

Our findings provide an indication that emergency healthcare-seeking may have had a drastic change amongst the population within the catchment area of ICHNT. The observed changes in ED attendances are likely driven by factors unique to the population seeking medical care at our institution. However, we also find that at a regional and national level, ED attendances decreased during the study period, which warrants further investigation. There are strong indications such a decrease in ED attendances have been driven by delayed and/or avoidance behaviours during the first wave of the COVID-19 epidemic, leading to an increase in preventable out-of-hospital deaths [9–12]. Overall, we find reduced ED attendance trends were maintained beyond a point when community-level COVID-19 case and death rates decreased. It remains unknown how the effective adaptation of alternative emergency health services outside of hospital led to alleviated pressure on EDs.

Going forward, it should be a public health priority to investigate optimal approaches to streamline emergency services, by creating safe pathways for urgent and emergency care outside of hospital settings. This will ensure high standards of care for both patients with COVID-19 and without COVID-19 can be maintained within EDs and hospitals.

## Supplementary Information

**Additional file 1 Figure S1. Daily positive cases and deaths of COVID-19 in London in 2020. Fig. S2. Monthly timeseries of attendances to ED services by region in 2020.** Red lines represent mean forecast from ARIMA model, with shadowed area representing the confidence interval (light purple 95% and dark purple 80%) and are compared against observed data (light blue line) from NHS Digital. **Fig. S3. Daily ED attendances to ICHNT by age, gender, and mode of arrival in 2020. Table S1. Timeline of Interventions in the UK. Fig. S4. Percent of ED attendances to ICHNT by geographic area of patient residence and method of arrival in 2020.** The distance is measured between the centre of the polygon containing the patient’s home address and either the location of St Mary’s or Charing Cross hospital. **Table S2. Number (%) of emergency department attendances by age group at Imperial College Healthcare NHS Trust in 2020. Table S3. Number (%) of emergency department attendances by gender at Imperial College Healthcare NHS Trust in 2020. Table S4. Number (%) of emergency department attendances by mode of arrival at Imperial College Healthcare NHS Trust in 2020. Table S5. Linear regression models for reduced number of ED attendances to Imperial College Healthcare NHS Trust by postcode of patient residence. Table S6**. **Historic (2015–2019) vs present deaths amongst emergency admissions by disease area at Imperial College Healthcare NHS Trust between March 12 and May 31**.

## Data Availability

Patient-level administrative data from ICHNT used for the conduction of this study are not publicly available due to Global Data Protection Regulations. Albeit these data were pseudonymized for analyses, it represents potentially identifiable and sensitive information. Publicly available data used, such as situation reports from NHS Digital, daily COVID-19 deaths in England count, shape files and the codes used for the analyses of these data can be accessed online (https://github.com/pabloperguz/aae_attendances). Reference to these data have been specified throughout the manuscript as relevant.

## Abbreviations

UK: United Kingdom
NHS: National Health Services
ARIMA: Autoregressive integrated moving average
CXH: Charing Cross Hospital
ED: Emergency department
ICHNT: Imperial College Healthcare NHS Trust
IMD: Index of multiple deprivation
J-IDEA: Abdul Lateef Jameel Institute for Disease and Emergency Analytics
RR: Risk ratio
SMH: St Mary’s Hospital
SNOMED: Systematised Nomenclature of Medicine Clinical Terms
SUS: Secondary Uses Services
