# Supplementary material for: The impact of the COVID-19 pandemic on patterns of attendance at emergency departments in two large London hospitals: an observational study
Source: BMC Health Serv Res. 2021 Sep 23;21:1008. doi: 10.1186/s12913-021-07008-9 (PMC8460185; doi:10.1186/s12913-021-07008-9)
Supplement: Supplementary file 1 — Additional file 1 Figure S1. Daily positive cases and deaths of COVID-19 in London in 2020. Fig. S2. Monthly timeseries of attendances to ED services by region in 2020. Red lines represent mean forecast from ARIMA model, with shadowed area representing the confidence interval (light purple 95% and dark purple 80%) and are compared against observed data (light blue line) from NHS Digital. Fig. S3. Daily ED attendances to ICHNT by age, gender, and mode of arrival in 2020. Table S1. Timeline of Interventions in the UK. Fig. S4. Percent of ED attendances to ICHNT by geographic area of patient residence and method of arrival in 2020. The distance is measured between the centre of the polygon containing the patient’s home address and either the location of St Mary’s or Charing Cross hospital. Table S2. Number (%) of emergency department attendances by age group at Imperial College Healthcare NHS Trust in 2020. Table S3. Number (%) of emergency department attendances by gender at Imperial College Healthcare NHS Trust in 2020. Table S4. Number (%) of emergency department attendances by mode of arrival at Imperial College Healthcare NHS Trust in 2020. Table S5. Linear regression models for reduced number of ED attendances to Imperial College Healthcare NHS Trust by postcode of patient residence. Table S6. Historic (2015–2019) vs present deaths amongst emergency admissions by disease area at Imperial College Healthcare NHS Trust between March 12 and May 31. [file 12913_2021_7008_MOESM1_ESM.docx]

**Supplementary appendix to: *The impact of the COVID-19 epidemic on all-cause attendances to emergency departments in two large London hospitals: an observational study***

*Auto Regressive Integrated Moving Average (ARIMA) model construction*

To forecast a counterfactual scenario of the number of ED attendances at Imperial College Healthcare NHS Trust (ICHNT), by England region and nationally, we created autoregressive integrated moving average (ARIMA) timeseries models. Albeit ICHNT data was pseudonymised and accessed through secure servers, only aggregated data (e.g., graphs, tables and summary statistics presented in this publication) was extracted, as records presented patient-level information and General Data Protection Regulations thus proscribe their public availability. Data for England regions, on the other hand was obtained from publicly available monthly situation reports between June 2015 and May 2020 by NHS Trusts.[1] The input file from this data source and an example source code for the ARIMA model for the London region can be accessed in https://github.com/MonkeyfaceMx/aae_attendances.git.

Trusts were categorised into four regions, London, North, South and Midlands. For each trust we extracted the number of ‘*type 1*’ attendances and emergency admissions per month and inputted into a timeseries format. In the English NHS context, a *type 1 ED* is a 24-hour hospital-based ED that is managed by clinical consultants.[1]

We used the time series data by region from June 2015 to December 2019 to parameterise and train the ARIMA models. Numerous ARIMA model selection algorithms have been described.[2] To minimise subjectivity in model selection criteria, we used the automatic forecasting algorithm function *auto.arima* from the R package *forecast*. This returns the automatic univariate ARIMA algorithm from Hyndman and Khandakar.[3] We assessed stationarity and differencing in the data with the augmented Dickey-Fuller test and Kwiatkowski, Phillips, Schmidt, & Shin test, respectively, as recommended in Hyndman and Athanasopoulos.[2]

All analyses were performed using R studio Version 1.2.5033. ***Figure S1*** shows the number of positive cases and deaths of COVID-19 in London. ***Figure*** ***S2*** below presents the outputs from the fitted forecast by England region in 2020. Disaggregated trends of ED attendances at ICHNT are presented in ***Figure S3*** and ***S4***. Please see main manuscript for discussion of these additional analyses.


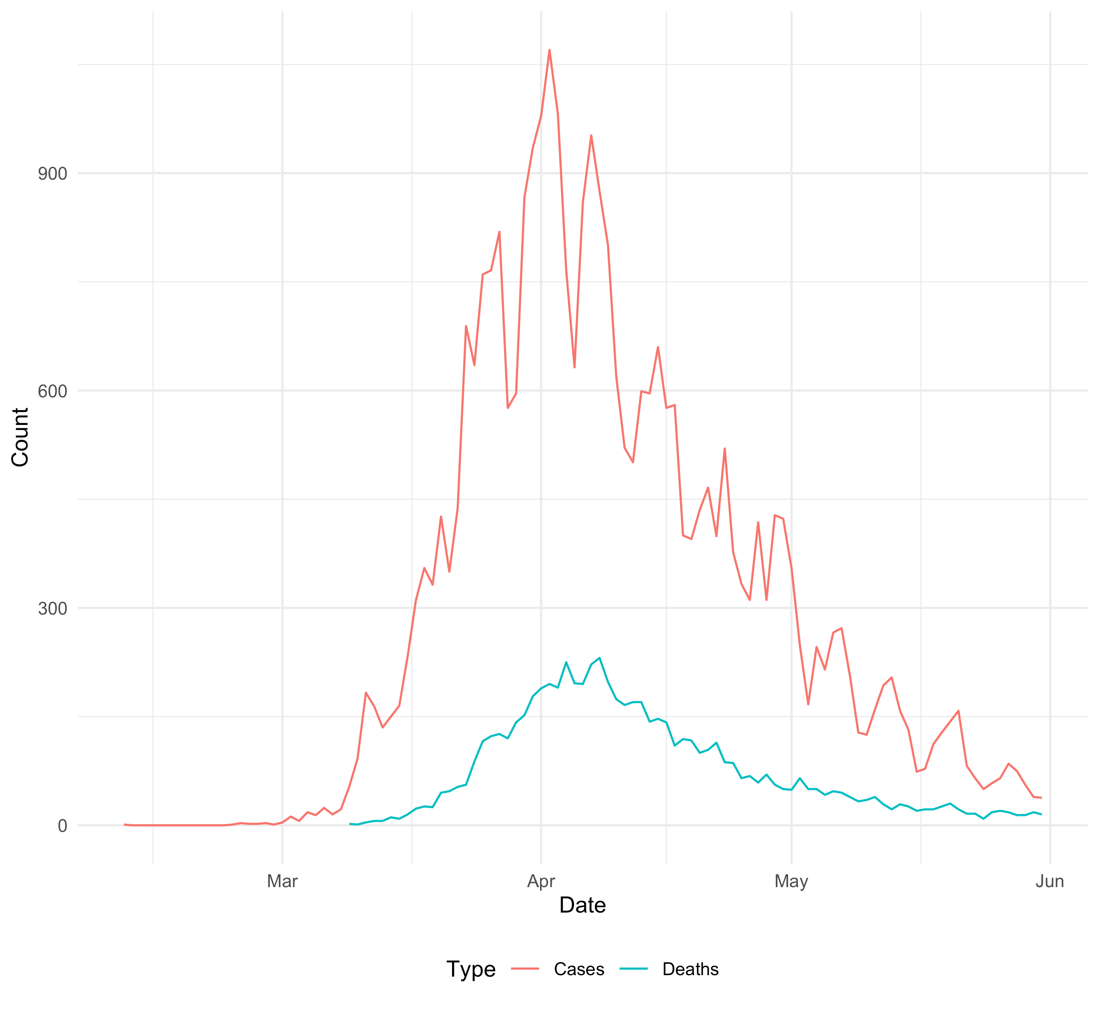


**Figure S1. Daily positive cases and deaths of COVID-19 in London in 2020.**

**Figure S2. Monthly timeseries of attendances to ED services by region in 2020.** Red lines represent mean forecast from ARIMA model, with shadowed area representing the confidence interval (light purple 95% and dark purple 80%) and are compared against observed data (light blue line) from NHS Digital.

a. Attendances by age


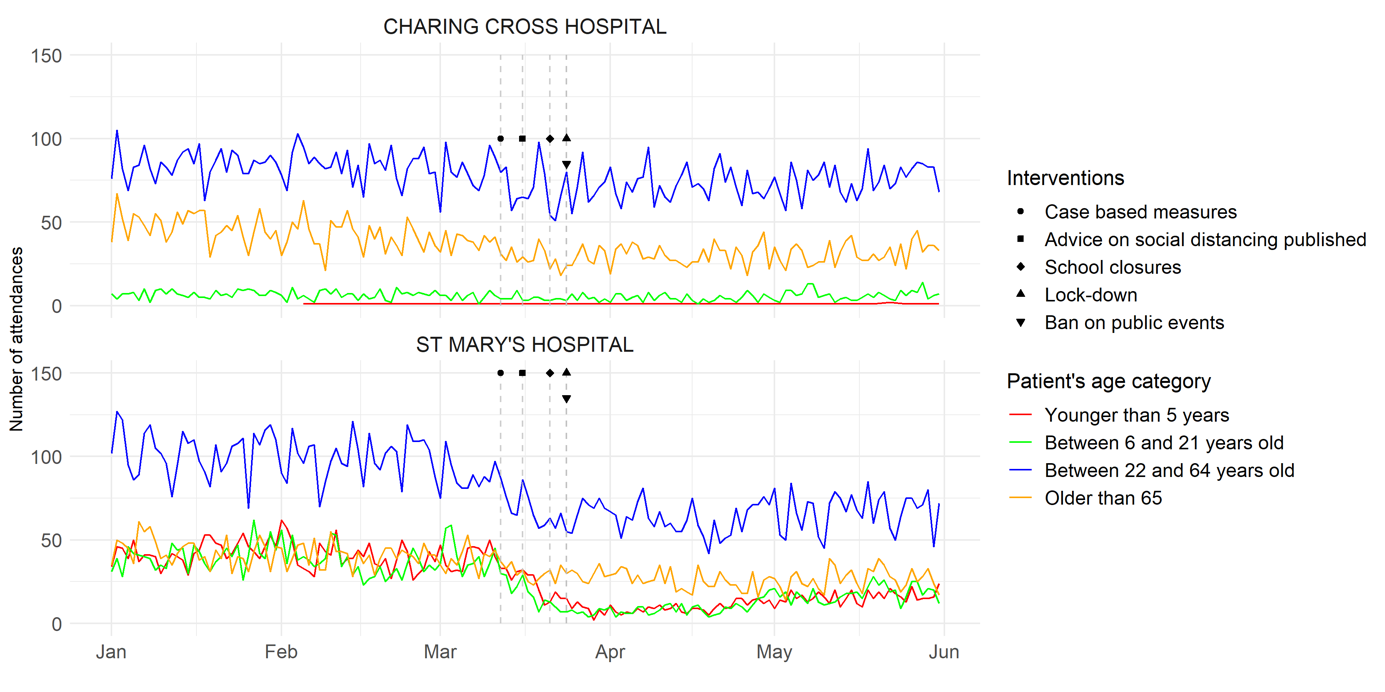


b. Attendances by gender


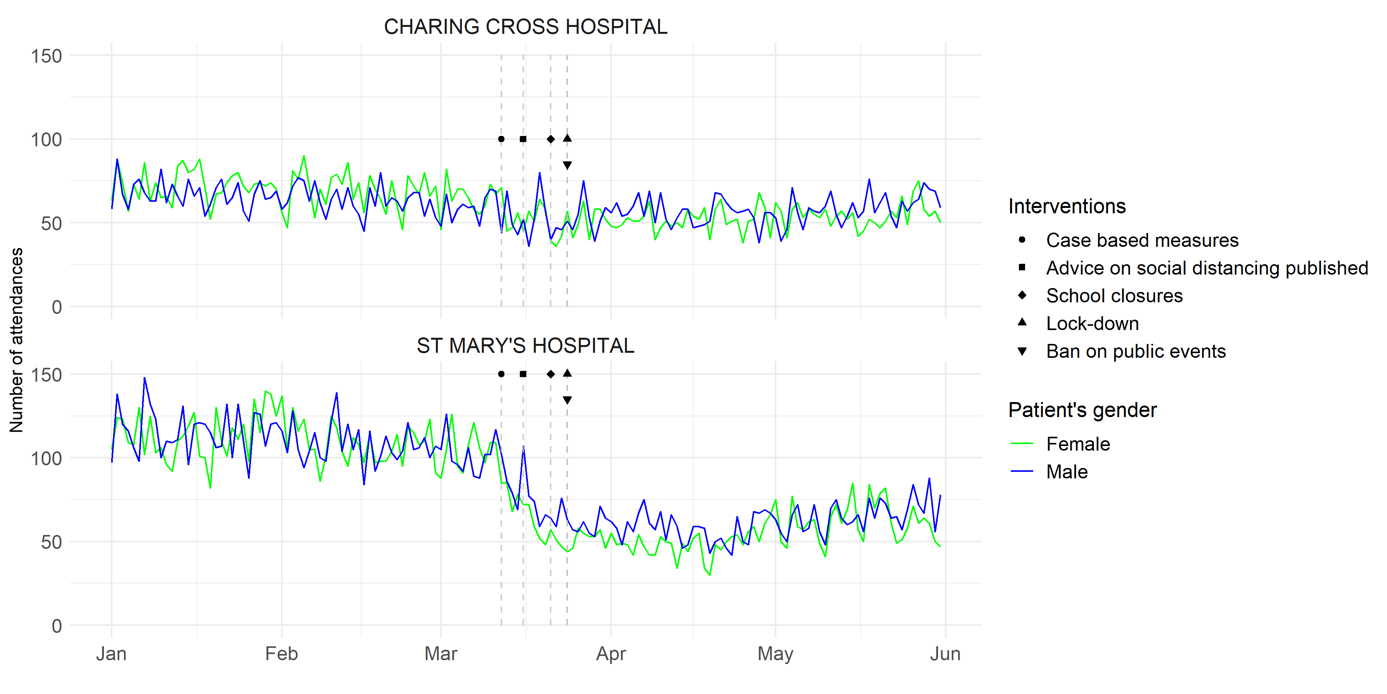


c. Attendances by mode of arrival


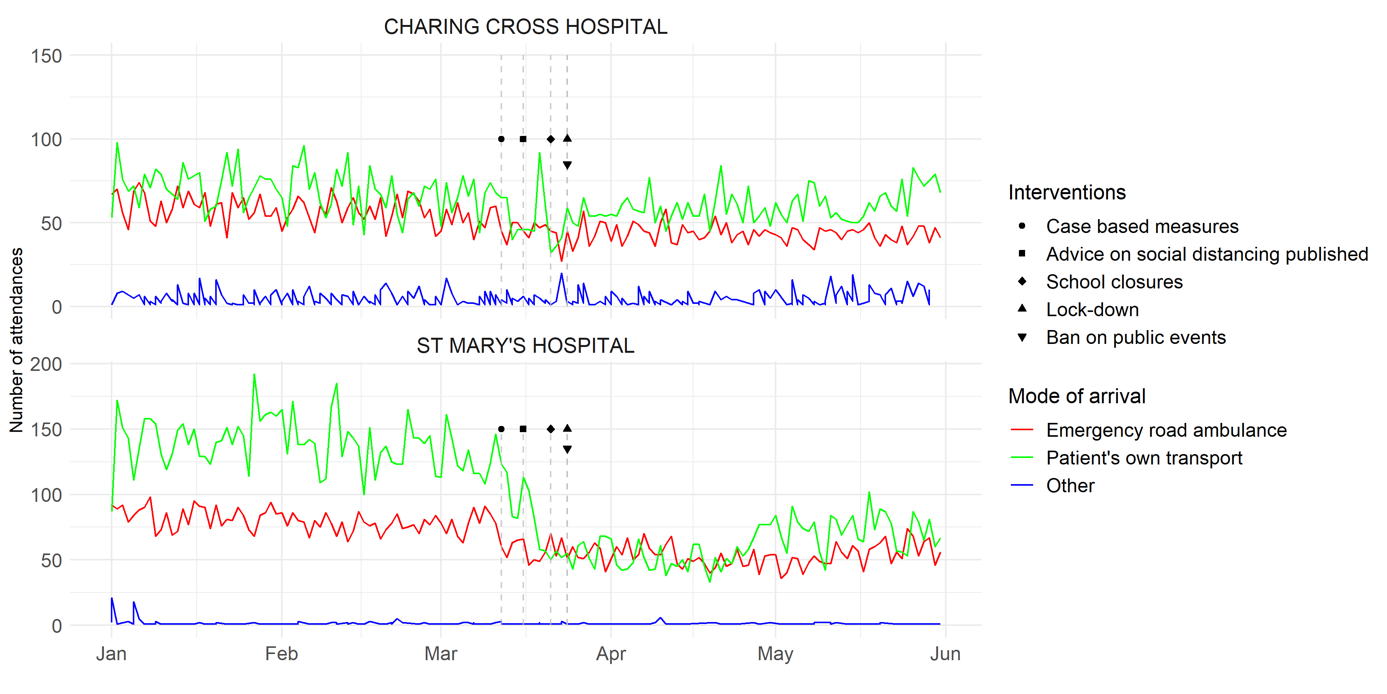


**Figure S3. Daily ED attendances to ICHNT by age, gender, and mode of arrival in 2020.**

**Table S1. Timeline of Interventions in the UK.**

| **Intervention** | **Definition** | **Dates** |
| --- | --- | --- |
| School closures | Nationwide school closure. Childminders, nurseries and sixth forms are told to follow the guidance. | 21-03-2020 |
| Public events banned | Implemented with lockdown. | 24-03-2020 |
| Lockdown ordered | Gatherings of more than 2 people not from the same household are banned and police enforceable. | 24-03-2020 |
| Social distancing encouraged | Advice to avoid pubs, clubs, theatres and other public institutions. | 16-03-2020 |
| Case based measures | Advice to self-isolate for 7 days if experiencing a cough or fever symptoms. | 12-03-2020 |

a. St Mary’s Hospital


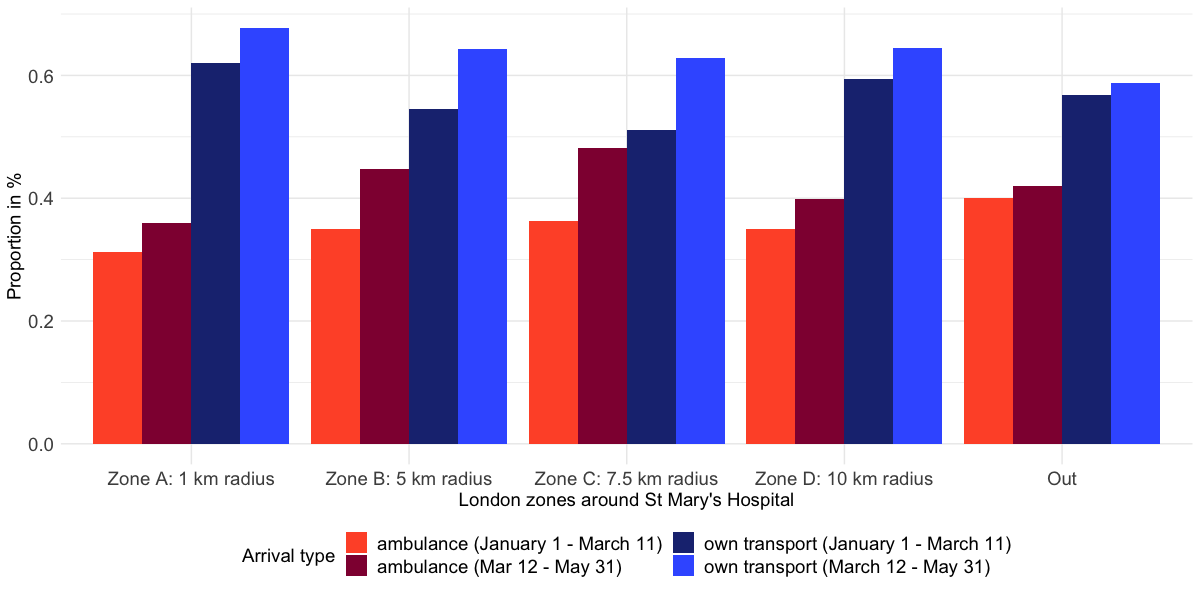


b. Charing Cross Hospital


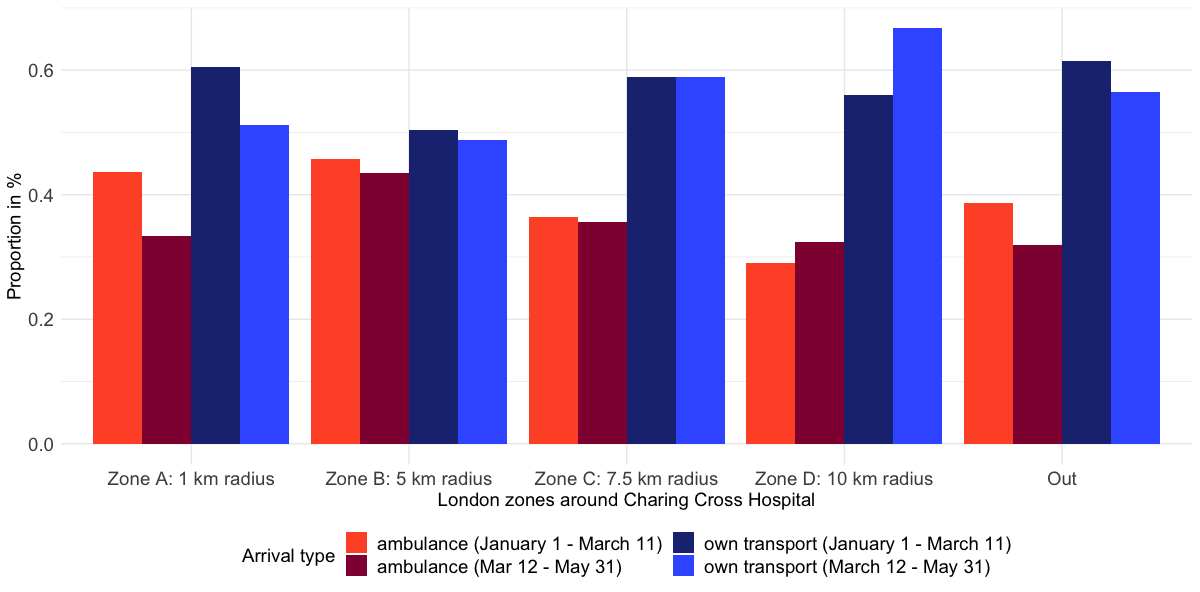


**Figure S4. Percent of ED attendances to ICHNT by geographic area of patient residence and method of arrival in 2020.** The distance is measured between the centre of the polygon containing the patient’s home address and either the location of St Mary’s or Charing Cross hospital.

**Table S2. Number (%) of emergency department attendances by age group at Imperial College Healthcare NHS Trust in 2020.**

|  | **Charing Cross Hospital** | | **St Mary’s Hospital** | |
| --- | --- | --- | --- | --- |
|  | *Jan 1 – March 11* | *March 12 – May 31* | *Jan 1 – March 11* | *March 12 – May 31* |
| **≤5** | 5 (0.1) | 24 (0.3) | 2,938 (18.8) | 1,146 (11.8) |
| **6 to 21** | 465 (4.9) | 422 (4.8) | 2,746 (17.5) | 1,093 (11.2) |
| **22 to 64** | 5,956 (62.5) | 5,925 (67.1) | 7,037 (44.9) | 5,310 (54.5) |
| **>65** | 3,109 (32.6) | 2,454 (27.8) | 2,947 (18.8) | 2,195 (22.5) |
| **Total** | **9,535 (100.0)** | **8,825 (100.0)** | **15,668 (100.0)** | **9,744 (100.0)** |

**Table S3. Number (%) of emergency department attendances by gender at Imperial College Healthcare NHS Trust in 2020.**

|  | **Charing Cross Hospital** | | **St Mary’s Hospital** | |
| --- | --- | --- | --- | --- |
|  | *Jan 1 – March 11* | *March 12 – May 31* | *Jan 1 – March 11* | *March 12 – May 31* |
| **Female** | 4,953 (51.9) | 4,273 (48.4) | 7,824 (49.9) | 4,579 (47.0) |
| **Male** | 4,582 (48.1) | 4,552 (51.6) | 7,841 (50.0) | 5,165 (53.0) |
| **Not specified** | 0 (0.0) | 0 (0.0) | 3 (0.0) | 0 (0.0) |
| **Total** | **9,535 (100.0)** | **8,825 (100.0)** | **15,668 (100.0)** | **9,744 (100.0)** |

**Table S4. Number (%) of emergency department attendances by mode of arrival at Imperial College Healthcare NHS Trust in 2020.**

*Includes prison/police transport, public transport and air ambulance.

|  | **Charing Cross Hospital** | | **St Mary’s Hospital** | |
| --- | --- | --- | --- | --- |
|  | *Jan 1 – March 11* | *March 12 – May 31* | *Jan 1 – March 11* | *March 12 – May 31* |
| Own transport | 4,949 (51.9) | 4,747 (53.8) | 9,841 (62.8) | 5,282 (54.2) |
| Road ambulance | 4,088 (42.9) | 3,535 (40.1) | 5,685 (36.3) | 4,383 (45.0) |
| Other* | 498 (5.2) | 543 (6.2) | 142 (0.9) | 79 (0.8) |
| **Total** | **9,535 (100.0)** | **8,825 (100.0)** | **15,668 (100.0)** | **9,744 (100.0)** |

*Linear regression models for reduced number of ED attendances at ICHNT*

To analyse the drivers of the changes in emergency admissions at ICHNT, we ran several linear regression models to identify any correlations. We looked at the distance from the patients’ postcode of residence to the hospital, five mutually exclusive zones, based on the distance of the centroid of the postcode area to the hospital of attendance, the population-weighted index of multiple deprivation (IMD) quintile as well as the mean number of historic attendances. In addition to running a regression model for all covariates, we also ran it including higher order terms such as the interaction between the historic mean times the postcode zones of the patient’s residence. Finally, we ran a stepwise regression which iteratively adds or removes predictors to find the best performing subset of covariates to achieve the lowest prediction error, in this case the highest AIC.

**Table S5. Linear regression models for reduced number of ED attendances to Imperial College Healthcare NHS Trust by postcode of patient residence.**

**Unscaled**

|  | SMH | CXH | SMH | CXH | SMH | CXH |
| --- | --- | --- | --- | --- | --- | --- |
| **(Intercept)** | -8.86  [-34.07, 16.35] | -144.00 ***  [-210.74, -77.25] | -0.04  [-29.83, 29.75] | 118.43  [-423.40, 660.27] | -0.71  [-3.24, 1.81] | -149.19 ***  [-213.45, -84.93] |
| **Distance (Km)** | -0.00  [-0.00, 0.00] | 0.00  [-0.00, 0.00] |  |  |  |  |
| **Mean historic attendances** | 0.58 ***  [0.57, 0.59] | 0.79 ***  [0.76, 0.81] | 0.57 ***  [0.55, 0.59] | 0.62 ***  [0.29, 0.95] | 0.58 ***  [0.57, 0.58] | 0.79 ***  [0.76, 0.81] |
| **Outermost zone** | 12.64  [-13.03, 38.31] | 141.02 ***  [74.66, 207.38] | -0.82  [-30.85, 29.21] | -118.29  [-656.63, 420.04] |  | 147.57 ***  [83.26, 211.87] |
| **Postcode zone B** ∆ | 7.75  [-14.12, 29.62] | 140.29 ***  [81.60, 198.99] | -8.50  [-39.70, 22.70] | -128.79  [-670.96, 413.37] |  | 140.75 ***  [82.29, 199.22] |
| **Postcode zone C** | 5.05  [-18.88, 28.99] | 132.85 ***  [67.06, 198.64] | -1.24  [-32.09, 29.61] | -121.37  [-663.62, 420.89] |  | 134.92 ***  [69.52, 200.32] |
| **Postcode zone D** | 11.18  [-13.34, 35.69] | 141.46 ***  [75.60, 207.31] | -1.42  [-32.26, 29.43] | -117.83  [-660.10, 424.44] |  | 144.66 ***  [79.42, 209.89] |
| **Weighed mean IMD quintile †** | -0.77  [-3.63, 1.08] | -1.94  [-7.06, 3.17] |  |  |  |  |
| **Mean historic attendances * outermost zone** |  |  | 0.09  [-0.07, 0.25] |  |  |  |
|  |  |  |  |  |  |  |
| **Mean historic attendances * Postcode zone B** |  |  | 0.02  [-0.00, 0.05] | 0.17  [-0.17, 0.50] |  |  |
| **Mean historic attendances * Postcode zone C** |  |  | -0.03  [-0.07, 0.01] | -0.03  [-0.43, 0.38] |  |  |
|  |  |  |  |  |  |  |
| **Mean historic attendances * Postcode zone C** |  |  | 0.05  [-0.10, 0.19] | 0.03  [-0.54, 0.59] |  |  |
| N | 132 | 122 | 132 | 132 | 132 | 122 |
| R2  AIC | 0.99  1081.535 | 0.99  1141.26 | 0.99  1073.721 | 0.99  1140.131 | 0.99  1073.598 | 0.99  1138.519 |
| *** p < 0.001; ** p < 0.01; * p < 0.05. | | | | | | |

† Weighed by total population in postcode.

∆ Intercept is postcodes within zone A (≤1,000 metres from the respective hospital).

*Emergency admissions by ICD-10 category*

To analyse changes in emergency admissions at ICHNT, we categorised patients into main disease areas as per ICD-10 codes. Only patients admitted into hospital from the ED were included in this analysis. Narrow disease categories were identified based on the first diagnosis recorded upon patient discharge/ death as follows:

- COVID-19: B97.2 or U07.1 or U07.2
- Acute respiratory conditions: J09-12 or J13-18 or J44.1 or J46
- Stroke I60-I69
- Acute coronary syndrome I20-I25
- Oncology C00-C97 or D01-D04
- Gastro & liver K00-K93
- Injuries T00-T98 or V01-V99 or W00-W99 or X00-X99 or Y00-Y89
- Genitourinary N00-N99

For specific cases where the main discharge/death diagnoses recorded were highly heterogeneous (i.e., ICD-10 codes for symptoms/signs [R00-R99] or for factors influencing contact with health services [Z00-Z99]) we aggregated admissions and deaths and classified them as *Other*.

Of note, whilst the mortality rate for the composite category ‘*Other*’ seemed to increase in 2020 compared to historic data (average in 2015-2019), mortality was both historically and in the present year very low. The actual number of deaths was thus very low and the statistical associations for a such a broad category are not conclusive. For risk of identifiability and in keeping with Global Data Protection Regulations, we do not present more granular data on this disease area (e.g., more granular areas only recorded 1 or 2 admissions and/or deaths in either period of time).

**Table S6**. **Historic (2015-2019) vs present deaths amongst emergency admissions by disease area at Imperial College Healthcare NHS Trust between March 12 and May 31.**

Historic data refers to the average of emergency admissions between the period from March 12 and May 31 for each year. Specific numbers by hospital are not presented due to risk of identifiability of individuals given low number of deaths and/or admissions.

*See text in section above for explanation of ICD-10 codes included

*Abbreviation: ICD-10, international classification of diseases; IRR, incident risk ratio*

|  | **2020** | | **Average 2015-2019** | |  |  |
| --- | --- | --- | --- | --- | --- | --- |
| **Disease area*** | **Deaths** | **Admissions** | **Deaths** | **Admissions** | **IRR** | **p-value** |
| Including COVID-19 | 523 | 6022 | 301 | 10406 | 2.84 (2.48, 3.26) | <0.01 |
| Non-COVID only | 164 | 4976 | 301 | 10406 | 1.13 (0.94, 1.37) | 0.19 |
| Acute respiratory conditions | 76 | 726 | 114 | 912 | 0.85 (0.65, 1.12) | 0.26 |
| Stroke | 25 | 188 | 30 | 258 | 1.13 (0.68, 1.86) | 0.64 |
| Acute coronary syndromes | 4 | 116 | 16 | 287 | 0.63 (0.22, 1.85) | 0.39 |
| Oncology | 20 | 263 | 50 | 484 | 0.75 (0.46, 1.24) | 0.26 |
| Gastro & liver | 6 | 366 | 15 | 829 | 0.91 (0.35, 2.32) | 0.84 |
| Injuries | 4 | 536 | 15 | 1471 | 0.73 (0.24, 2.20) | 0.58 |
| Genitourinary | 7 | 308 | 14 | 834 | 1.35 (0.55, 3.30) | 0.52 |
| Other | 22 | 1474 | 20 | 3552 | 2.63 (1.44, 4.80) | <0.01 |

**References**

1. NHS England. A&E Attendances and Emergency Admissions. 2020. https://www.england.nhs.uk/statistics/statistical-work-areas/ae-waiting-times-and-activity/. Accessed 9 Jun 2020.

2. Hyndman RJ, Athanasopoulos G. Forecasting: Principles and Practice. 2nd edition. Melbourne, Australia: OTexts; 2018. https://otexts.com/fpp2/. Accessed 1 Jul 2020.

3. Hyndman RJ, Khandakar Y. Automatic Time Series Forecasting: The forecast Package for R. J Stat Soft. 2008;27. doi:10.18637/jss.v027.i03.
